# Genetically determined height was associated with lung cancer risk in East Asian population

**DOI:** 10.1002/cam4.1557

**Published:** 2018-05-23

**Authors:** Lu Wang, Mingtao Huang, Hui Ding, Guangfu Jin, Liang Chen, Feng Chen, Hongbing Shen

**Affiliations:** ^1^ Department of Epidemiology and Biostatistics School of Public Health Nanjing Medical University Nanjing China; ^2^ Jiangsu Key Lab of Cancer Biomarkers, Prevention and Treatment Collaborative Innovation Center of Cancer Medicine Nanjing Medical University Nanjing China; ^3^ Department of Thoracic Surgery First Affiliated Hospital of Nanjing Medical University Nanjing China

**Keywords:** Adult height, East Asian population, Lung cancer, Mendelian randomization

## Abstract

The association between adult height and risk of lung cancer has been investigated by epidemiology studies, but the results are inconsistent. Mendelian randomization (MR) analyses with individual‐level data from two genome‐wide association studies, including a total of 7127 lung cancer cases and 6818 controls, were carried out to explore whether adult height is causally associated with risk of lung cancer. A weighted genetic risk score (wGRS) was created based on genotypes of 101 known height‐associated genetic variants. Association between the wGRS and risk of lung cancer was analyzed by logistic regression for each study separately. The combined effect was calculated using fixed effect meta‐analysis. MR analyses showed that increased risk of lung cancer (OR = 1.19, 95%CI: 1.05‐1.35, *P *=* *0.006) associated with taller genetically determined height. Compared with individuals in the lowest tertile of the height‐associated wGRS, those in the highest tertile had 1.10‐fold (95% CI: 1.01‐1.20) increased risk of developing lung cancer. Sensitivity analyses excluding BMI‐associated genetic variants demonstrated consistent association. Our study suggested that genetically taller height was associated with increased risk of lung cancer in East Asian population, indicating that increasing height may have a causal role in lung cancer carcinogenesis.

## 1 INTRODUCTION

Lung cancer is one of the leading causes of cancer morbidity and mortality worldwide.^1^ It is estimated that there were 1.8 million incident cases of lung cancer and 1.6 million cause‐specific deaths, accounting for nearly one‐fifth of total cancer deaths in 2013.^2^ As known, lung cancer is a multifactorial disease involving both environmental and genetic factors. Tobacco smoking is the main risk factor for lung cancer, relating to approximately 90% of lung cancer cases.^3^ Other known risk factors for lung cancer include exposure to occupational and environmental carcinogens such as asbestos and outdoor pollution.^4^, ^5^

Human anthropometric indicators are associated with multiple diseases, including cancers. Over the past decade, plenty of epidemiological studies have investigated the associations between body‐mass index (BMI) and cancer risk^6^, ^7^; however, the relationship between adult height and cancer risk has received much less attention. Adult height is a complex and highly heritable trait that is determined by both genetic and environmental factors. The heritability of height has been estimated to be up to 80%‐90%.^8^, ^9^, ^10^ Nutrition, diseases, as well as socioeconomic status, are important environmental factors that might affect body height in adulthood.^11^ Moreover, height measurement is noninvasive, cost‐efficient, and accurate in population‐based studies, which makes adult height become a potential tool for monitoring health conditions.^12^

Several previous studies have investigated the association of adult height with risk of lung cancer, but the results are inconsistent. A Korea cohort study reported each 5‐cm increment in height was associated with increased risk of lung cancer.^13^ Similar associations were also recorded in two recent meta‐analyses^14^, ^15^; however, the results from Million Women Study in UK did not find significant associations between height and risk of lung cancer.^16^ It remains unclear whether the observed association reflects a causal effect of adult height on lung cancer, or is due to confounding or biases inherent in conventional epidemiological studies.

Mendelian randomization (MR) is a technique of using genetic variants to estimate the causal effect of a modifiable risk factor from observational data.^17^ As genotypes generate through alleles randomly assort at gamete formation and segregate randomly at conception, associations between genotypes and outcome are not generally confounded by environmental factors and therefore can avoid reverse causation.

In this study, we used MR approach to assess the association between height and risk of lung cancer using individual‐level data from 13 945 subjects of East Asian population. We derived a weighted genetic risk score (wGRS) comprising 101 height‐related single nucleotide polymorphisms (SNP) identified by previous genome‐wide association studies (GWAS) in East Asian‐ancestry populations and analyzed whether there is a causal relationship between height and risk of lung cancer.

## 2 MATERIALS AND METHODS

### 2.1 Study subjects

We used two existing data from previously published lung cancer GWAS studies, that is, the Nanjing Medical University (NJMU) and the Female Lung Cancer Consortium in Asia (FLCCA). The details of these two studies were described elsewhere.^18^, ^19^, ^20^ In brief, the NJMU study included 2331 cases and 3077 controls from Nanjing, Shanghai, Beijing, and Wuhan in China. The FLCCA study was obtained via the database of Genotypes and Phenotypes (dbGAP), and included 4922 cases and 3959 controls from mainland China, South Korea, Japan, Singapore, Taiwan and Hong Kong. For the FLCCA study, we also excluded the overlapped subjects between FLCCA GWAS and NJMU GWAS, thus, 4796 cases and 3741 controls were included in following analyses. Adult height data of the controls were only available in the NJMU Nanjing Study. Height measurement followed standard procedure and was measured to the nearest 0.1 cm. All study participants provided their written informed consent, and the study protocols were approved by the relevant Institutional Review Boards. The demographic characteristics of study population are summarized in Table S1. In total, our analysis consisted of 7127 lung cancer cases and 6818 controls from samples of East Asian descent. The lung cancer cases included 4773 lung adenocarcinoma, 1482 lung squamous cell carcinoma and 872 other lung cancer types.

### 2.2 Genotype imputation

Full details of the genotyping, quality control and imputation have been reported previously.^18^, ^19^, ^20^ Briefly, the NJMU GWAS was conducted using Affymetrix Genome‐Wide Human SNP Array 6.0 with standard GWAS quality‐control procedures. The FLCCA GWAS was conducted using Illumina Human610_Quadv1_B and Human660W‐Quad_v1_A whole genome genotyping array and downloaded from the database of dbGaP database (dbGaP Study Accession: phs000716.v1.p1). After initial quality control, we excluded individuals with low call rates (<95%), familial relationships and extreme heterozygosity rates, and removed SNPs with low call rates (<95%), minor allele frequency (MAF) <5% and *P *<* *1 × 10^−6^ for the Hardy‐Weinberg equilibrium test in controls. Genotype imputation was performed with IMPUTE2 (V.2.2.2) software using the 1000 Genomes Project Phase 3 as a reference. Population structure was evaluated by principal components analysis using the software package EIGENSTRAT 3.0 for the NJMU study and the FLCCA study separately.

### 2.3 Identification of SNPs associated with height

We selected SNPs associated with adult height from three previously published GWAS studies among individuals of East Asian ancestry.^21^, ^22^, ^23^ We identified 110 SNPs showing genome‐wide significance level (*P *<* *5 × 10^−8^) in East Asian population. To infer adult height more effectively, we further included 26 additional SNPs which were previously reported to be associated with height in populations of European ancestry and replicated with significance level (*P *<* *1 × 10^−5^) in East Asian studies. Two variants, rs234886 and rs12680655, were not available in our lung cancer GWAS data and were replaced by high linkage disequilibrium (LD) SNPs, rs2237886 and rs733254 (*r* ^2 ^> 0.9 in 1000 Genomes Project Phase 3 East Asian population). Thirty‐five SNPs were in moderate LD (*r* ^2 ^> 0.1) with other SNPs based on 1000 Genomes Project Phase 3 data and then excluded in subsequent analyses. Finally, a total of 101 independent height‐related SNPs were used as instrumental variables in our study. In sensitivity analyses, we further excluded SNPs which were associated with BMI or had moderate LD (*r* ^2 ^> 0.1) with BMI‐related SNPs in East Asian population. Thirty‐seven BMI‐related SNPs were selected from a recently published BMI MR study.^24^

### 2.4 MR assumptions

The genetic variants used as instrumental variables in MR need to meet three assumptions^25^: (1) the genetic variants are associated with the exposure; (2) the genetic variants affect the outcome only via the exposure; (3) the genetic variants are not associated with any confounders that may affect the exposure‐outcome association. Based on the second assumption, a SNP’s association with height should be proportional to its association with cancer risk. Therefore, we assessed the potential violation (pleiotropic effect) of the latter two assumptions using pleiotropy test in “gtx” package (v0.0.8) in R software. Six SNPs in NJMU study and three SNPs in FLCCA study were excluded for further analyses (Tables S2 and S3). Finally, 95 SNPs in NJMU study and 98 SNPs in FLCCA study were used in our study.

### 2.5 Statistical analysis

We calculated the wGRS for height‐related associated variants as an instrumental variable. To create wGRS for the i‐th subject from the height‐associated variants the following formula was used:

$${\text{GRS}}_{i}=\sum_{\left(j=1\right)}^{9}w_{j}x_{\mathrm{ij}}$$

Here, *x*_*ij*_ is the number of risk alleles for the *j*‐th SNP in the *i*‐th subject (*x*_*ij*_ = 0, 1 or 2 for wild‐type homozygous, heterozygous, or homozygous for the effect allele associated with taller height) and *w*_*j*_ is the weight or coefficient for the *j*‐th SNP. The weights or coefficients were the height‐associated β‐estimates scaled to the *z*‐score‐transformed height per tall allele, obtaining from the previously published GWAS studies. The wGRS could be used as an instrumental variable to represent z‐score‐transformed height in individual level. A higher wGRS indicates taller height, whereas a lower wGRS for shorter height, using association estimate for the tall allele. The effect of wGRS was estimated separately for two studies using logistic regression adjusted for age, sex, pack‐years, and first principal component in NJMU study and adjusted for age, and three principal components in FLCCA study. The combined effect and heterogeneity of height‐associated wGRS on lung cancer was calculated by meta‐analysis.

Besides the wGRS approach, we estimated the effect of height on risk of lung cancer using inverse‐variance weighting (IVW) method, which is a summary data based on MR method. Summary association estimates were used in IVW to calculate causal effects, which was described in greater detail by Burgess et al^26^ The potential causal association between height and risk of lung cancer was modeled using height‐related SNPs’ β‐estimates obtaining from published GWAS studies. The Cochran’s *Q* test was used to assess the pleiotropic effect for the genetic variants. The IVW method was conducted in R software using “gtx” package (v0.0.8).

To further examine the relationship between height and lung cancer, we categorized height‐related wGRS into three groups based on its tertile distribution in all participants. We accessed potential non‐linear trends between height and risk of lung cancer using a restricted cubic spline analysis.^27^ Same statistic methods with wGRS were used to estimate the effect and heterogeneity of SNPs. To identify common biologic processes that might explain the association between height and lung cancer, we performed KEGG pathway analysis using R package “clusterProfiler” (v3.0.5). The genes that 101 height‐associated SNPs locate in or nearest were involved in pathway analysis (Table S2).

All statistical analyses were performed by R version 3.3.1. All statistical tests were two‐sided with *P *<* *0.05 considered statistically significant.

## 3 RESULTS

### 3.1 Height‐associated variants and lung cancer

The associations between 101 height‐related SNPs and lung cancer are shown in Table S2. Most of the height‐related SNPs were not significant in both two studies. However, there were 15 SNPs in NJMU study and 13 SNPs in FLCCA study had nominally significant associations (*P *<* *0.05), and only one SNP, rs174547, in NJMU study survived Bonferroni correction (*P *<* *4.95 × 10^−4^). However, rs174547 was removed after pleiotropy test.

### 3.2 wGRS and observed height in controls

**Figure 1:**
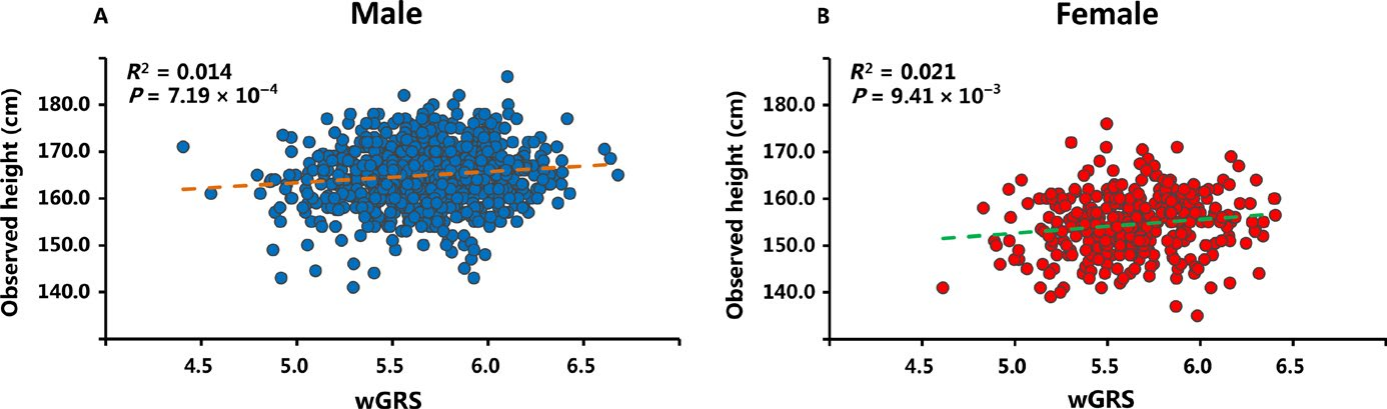
Relation of height‐associated variants wGRS with observed height from 1146 controls in the NJMU study Nanjing samples. Best‐fit lines (dash line) are drawn for the relationship of measured height with height‐associated variants wGRS for (A) male (N = 820) and (B) female (N = 326)

We first evaluate the relationship between observed height and the wGRS in 1146 controls. As shown in Figure 1, the increasing wGRS was significantly associated with higher observed height in both male (*r* ^2 ^= 0.014, *P *=* *7.19 × 10^−4^) and female (*r* ^2 ^= 0.021, *P *=* *9.41 × 10^−3^) participants, the estimated effect was in the positive direction (male β estimate = 2.37, female β estimate = 2.92). Therefore, this provides evidence that the wGRS of height‐associated variants has utility in predicting adult height in East Asian populations.

### 3.3 Genetically determined height associates with risk of lung cancer

As shown in Table 1, the meta‐analysis indicated that higher genetically predicted height was associated with increased risk of all lung cancer (OR = 1.19, 95% CI = 1.05‐1.35, *P *=* *0.006). The significant associations were also found in lung adenocarcinoma (OR = 1.18, 95% CI = 1.03‐1.36, *P *=* *0.020) and lung squamous cell carcinoma (OR = 1.29, 95% CI = 1.04‐1.61, *P *=* *0.021). The results of two studies are presented in Table S4. No significant heterogeneity was observed among two studies. When modeled as a tertile of height‐associated wGRS, wGRS was significantly associated with an increased risk of all lung cancer (per tertile OR = 1.05, 95% CI = 1.01‐1.09, trend *P *=* *0.028) and lung squamous cell carcinoma (per tertile OR = 1.08, 95% CI = 1.01‐1.17, trend *P *=* *0.035); however, not significant in lung adenocarcinoma (per tertile OR = 1.04, 95% CI = 0.99‐1.09, trend *P *=* *0.094). Compared with individuals in the lowest tertile of wGRS, those in the highest tertile had a 1.10‐fold (95% CI = 1.01‐1.20, *P *=* *0.029), 1.09‐fold (95% CI = 0.99‐1.19, *P *=* *0.094), and 1.18‐fold (95% CI = 1.01‐1.37, *P *=* *0.035) increased risk of developing all lung cancer, lung adenocarcinoma and lung squamous cell carcinoma, respectively (Table 2). Additionally, we did not observe a significant non‐linear association (*P*_non‐linear _> 0.05) between the height‐related wGRS and risk of lung cancer among two studies (Figure S1), suggesting a potential linear effect of genetic determined height on risk of lung cancer. Furthermore, we estimated the potential causal effect of height on lung cancer using the MR IVW method with summary statistics of each height‐related SNPs. Compared with that using the wGRS method, similar associations between height and risk of lung cancer were observed among all lung cancer, lung adenocarcinoma and lung squamous cell carcinoma (Table 1).

**Table 1:** Effect estimates for associations of genetic instruments with height and lung cancer risk

Genetic instruments	Lung adenocarcinoma		Lung squamous cell carcinoma		All lung cancer	
	OR (95% CI)	*P*	OR (95% CI)	*P*	OR (95% CI)	*P*
wGRS^a^	1.18 (1.03, 1.36)	0.020	1.29 (1.04, 1.61)	0.021	1.19 (1.05, 1.35)	0.006
IVW^a^	1.18 (1.03, 1.36)	0.018	1.30 (1.04, 1.62)	0.021	1.19 (1.05, 1.35)	0.006
Unweighted GRS	1.01 (1.00, 1.01)	0.007	1.01 (1.00, 1.02)	0.029	1.01 (1.00, 1.01)	0.003
wGRS unrelated to BMI^b^	1.15 (1.00, 1.33)	0.046	1.26 (1.01, 1.57)	0.045	1.16 (1.02, 1.32)	0.020
IVW unrelated to BMI^b^	1.16 (1.00, 1.33)	0.045	1.26 (1.00, 1.57)	0.046	1.16 (1.03, 1.32)	0.019
Heterogeneity^c^	*I* ^2 ^= 19.3%	0.266	*I* ^2 ^= 0	0.821	*I* ^2 ^= 15.8%	0.276

a The OR and *P* value were calculated from meta‐analysis of two studies.

b The OR and *P* value were calculated with SNPs after excluding BMI‐related SNPs.

c Heterogeneity test was conducted for wGRS of two studies.

**Table 2:** Associations of categories of genetic risk score (wGRS) predicting between height and lung cancer risk

wGRS categories	Lung adenocarcinoma^a^		Lung squamous cell carcinoma^a^		All lung cancer^a^	
	OR (95% CI)	*P*	OR (95% CI)	*P*	OR (95% CI)	*P*
Q1	Ref.		Ref.		Ref.	
Q2	1.06 (0.97, 1.17)	0.209	1.08 (0.92, 1.25)	0.345	1.08 (1.00, 1.18)	0.062
Q3	1.09 (0.99, 1.19)	0.094	1.18 (1.01, 1.37)	0.035	1.10 (1.01, 1.20)	0.029
*P* for Trend		0.094		0.035		0.028

a The OR and *P* value were calculated from meta‐analysis of two studies.

### 3.4 Sensitivity analyses

To determine whether the analyses were robust to the choice of weights used in the genetic risk score calculation, we analyzed the causal association of height and risk of lung cancer using an unweighted GRS. As expected, the association of unweighted GRS remained statistically significant (Table 1), though, the effect size was attenuated. Nor did the results of wGRS change substantially when we additionally excluded the BMI‐related SNPs which might interfere the genetic risk of adult height (Table 1).

### 3.5 Pathway analysis

To further clarify the potential mechanism of genetically determined height in the development of cancer, we performed pathway analysis. A total of eight significant enriched KEGG pathways (*Q* value < 0.05) were identified (Table 3). The most significant enriched pathway was transforming growth factor (TGF)‐β signaling pathway, an essential biological pathway of growth and development processes. We also found another two pathways, transcriptional misregulation in cancer and proteoglycans in cancer, directly related to cancer. Most of the significant enriched KEGG pathways, such as TGF‐β, MAPK, Hippo and Hedgehog signaling pathway were important in both development and carcinogenesis.

**Table 3:** Biologic pathways identified by KEGG of height‐associated genes

KEGG ID	Description	GeneRatio^a^	*P* value	*Q* value^b^	Genes
hsa04350	TGF‐beta signaling pathway	0.14	8.30 × 10^−6^	0.001	*LTBP1/RBL1/MYC/TGFB2/BMP2/BMP6*
hsa04913	Ovarian steroidogenesis	0.09	1.96 × 10^−4^	0.011	*IGF1R/BMP6/IGF1/CYP19A1*
hsa05202	Transcriptional misregulation in cancer	0.14	6.94 × 10^−4^	0.025	*MEF2C/HMGA2/MYC/IGF1R/IGF1/SUPT3H*
hsa05205	Proteoglycans in cancer	0.14	1.10 × 10^−3^	0.030	*CCND1/MYC/TGFB2/IGF1R/IGF1/PTCH1*
hsa04010	MAPK signaling pathway	0.16	1.46 × 10^−3^	0.032	*MEF2C/MYC/TGFB2/MAP3K3/IGF1R/IGF1/GNA12*
hsa04390	Hippo signaling pathway	0.12	1.96 × 10^−3^	0.035	*CCND1/MYC/TGFB2/BMP2/BMP6*
hsa04630	Jak‐STAT signaling pathway	0.12	2.44 × 10^−3^	0.035	*CCND1/GHR/SOCS2/MYC/STAT2*
hsa04340	Hedgehog signaling pathway	0.07	2.56 × 10^−3^	0.035	*CCND1/HHIP/PTCH1*

a The gene ratio is the proportion of the genes in the KEGG pathway that were part of the input list for the height‐related genes.

b The *Q* value was calculated for determining the false discovery rate.

## 4 DISCUSSION

In this study, we applied the MR approach to investigate the relationship between genetically determined height and risk of lung cancer. We found that the increase of height wGRS was roughly linear associated with increased risk of lung cancer, as well as lung adenocarcinoma and lung squamous cell carcinoma subtypes. The findings were robust in analyses used MR IVW method, an unweighted GRS method, or additionally excluded BMI‐associated genetic variants.

Several large prospective cohort studies have assessed the associations between adult height and risk of cancers. Increased adult height has been reported positively associated with the risk of all cancer combined and several site‐specific cancers.^13^, ^16^, ^28^, ^29^ Green et al^16^ observed significant positive associations of adult height with up to 10 cancer types based on UK Million Women Study, but not including lung cancer. By contrast, Sung et al^13^ observed a significant positive association of adult height with lung cancer in a male Korean population. The inconsistent result may be caused by different genetic background between populations or the underlying confounding in observational studies. A recent meta‐analysis concluded that per 10‐cm increase in height was positively associated with 6% increased risk of lung cancer. Modest association between genetically determined height and risk of lung cancer, based on European population, was reported using MR analysis. Moreover, the increased risk for lung adenocarcinoma appears to be stronger than lung squamous cell carcinoma.^14^ Our results are in line with previous observational study in Korean population and the height MR study. For the analysis presented here, we provide novel evidence of potential causal effect of adult height on risk of lung cancer in East Asian population.

Although numerous previous studies have proved that height may increase cancer risk, the underlying biological mechanisms are still unclear. Adult height is not only determined by a large proportion of genetic factors, but also affected by environmental factors such as nutrition during early life and adolescence, infections and diseases. One possible common mechanism is that hormone levels, especially growth factors like insulin‐like growth factors (IGFs), may play an important role in height‐related increasing cancer risk.^30^, ^31^ IGF‐I levels in childhood and adolescence are strongly related to skeletal growth,^32^ what is more, circulating levels of IGF‐1 has been identified as a risk factor of breast, prostate and colorectum cancer.^33^, ^34^, ^35^, ^36^ Another possible explanation is that taller people have more cells, thus a greater opportunity for mutations leading to malignant transformation.^37^, ^38^ Shared genetic factors may also be a possible explanation. Studies have found that genes related to increased height are also linked with oncogenic pathways, such as p53, c‐Myc, and SMAD3.^39^ Several height‐related SNPs have also been reported to be associated with risk of testicular and prostate cancer.^40^ In our analysis, we found height‐related genes were enriched in eight KEGG pathways which play important roles in both development and carcinogenesis. TGF‐β, MARK and Hedgehog signal pathway have been identified by GWAS to be associated with adult height.^21^, ^40^, ^41^ Meanwhile, the pathways mentioned above are also involved in the pathogenesis of cancer.^42^, ^43^, ^44^ For instance, activation of the TGF‐β signaling pathway induces potent cell‐cycle arrest in healthy noncancerous cells and in early‐stage cancerous cells, suggesting that this pathway plays a prominent role in tumor suppression. Therefore, height and cancer development may share the same genetic susceptibility.

Mendelian randomization is an alternative way to estimate the causal effect of exposure of interest on diseases. Compared with observational studies, MR can avoid both reverse causation bias and potential confounding bias. MR analysis can only be used to infer causal effect correctly when three key assumptions are satisfied (Methods). For the first assumption, we selected genetic variants which showed genome‐wide significant association with height in East Asian population or those identified in European population, but can be validated in East Asian population. In accordance with the strict selection criteria, the associations between SNPs and adult height are reliable. For the second assumption, it is possible that height‐related genetic variants are associated with other risk factors that influence the cancer development. That is, those SNPs have pleiotropic effect and the effect of those SNPs on the risk of height that are independent of any effect through height. After excluding those SNPs failed in pleiotropy test using “gtx” package, the pleiotropy test of remained SNPs was not significant, indicating that there was no pleiotropic effect to disturb the causal inference in MR analysis. For the third assumption, we did not observe significant associations of wGRS with potential confounding, like age, gender, or pack‐year. Therefore, we believe the MR analysis performed in current study basically satisfied the three key assumptions and the estimate of causal effect was highly reliable.

To our knowledge, this is the first study to investigate association between adult height and risk of lung cancer in East Asian population. The main strength of our study is that we used MR method to analyze genetic determined height and lung cancer. Using MR method can avoid the reverse causation bias and confounding bias due to traditional epidemiological studies. In addition, we test the linear trend of genetic determined height based on individual wGRS. We also examined the association in two main lung cancer subtypes, lung adenocarcinoma and lung squamous cell carcinoma. However, our study has several limitations. Firstly, all the GWAS identified SNPs only explained a small amount of variance in adult height. Secondly, our study included two previously published GWAS data, one from Chinese population and another from nonsmoking women in Asian. There might be geographic difference between the two populations, although the heterogeneity test was not significant. The subgroup analysis of gender and smoke status was not conducted because one of the studies involved only women and nonsmokers. Thus, female take a large proportion of all samples, the results might not be representative.

In summary, our findings from the MR analysis provide strong evidence that increasing height is associated with increased risk of lung cancer in East Asian population. It is shown that height may be a causal risk factor in lung cancer development. Our study suggests the biologic pathways that genetically determined adult height may also involve in the etiology of lung cancer. However, further investigations are needed to clarify the underlying mechanism.

## CONFLICT OF INTEREST

The authors have no conflicts of interest to declare.
