# Supplementary material for: Genetically determined height was associated with lung cancer risk in East Asian population
Source: Cancer Med. 2018 May 23;7(7):3445–52. doi: 10.1002/cam4.1557 (PMC6051217; doi:10.1002/cam4.1557)
Supplement: Supplementary file 1 [file CAM4-7-3445-s001.docx]

**Supplementary Table 1.** Characteristics of cases and controls

| **Characteristics** | **Overall** | |  | **NJMU GWAS** | |  | **FLCCA GWAS** | |
| --- | --- | --- | --- | --- | --- | --- | --- | --- |
|  | **Cases** | **Controls** |  | **Cases** | **Controls** |  | **Cases** | **Controls** |
| All | 7127 | 6818 |  | 2331 | 3077 |  | 4796 | 3741 |
| Age (%) |  |  |  |  |  |  |  |  |
| <60 | 3519 (49.38%) | 3452 (50.63%) |  | 1111 (47.66%) | 1429 (46.44%) |  | 2408 (50.21%) | 2023 (54.08%) |
| ≥60 | 3608 (50.62%) | 3366 (49.37%) |  | 1220 (52.34%) | 1648 (53.56%) |  | 2388 (49.79%) | 1718 (45.92%) |
| Gender (%) |  |  |  |  |  |  |  |  |
| Male | 1711 (24.01%) | 2086 (30.60%) |  | 1711 (73.40%) | 2086 (67.79%) |  | - | - |
| Female | 5416 (75.99%) | 4732 (69.40%) |  | 620 (26.60%) | 991 (32.21%) |  | 4796 (100%) | 3741 (100%) |
| Smoking (%) |  |  |  |  |  |  |  |  |
| Never | 5621 (78.87%) | 5509 (80.80%) |  | 825 (35.39%) | 1768 (57.46%) |  | 4796 (100%) | 3741 (100%) |
| Smoker | 1506 (21.13%) | 1309 (19.20%) |  | 1506 (64.61%) | 1309 (42.54%) |  | - | - |
| Histology (%) |  |  |  |  |  |  |  |  |
| Squamous cell carcinoma | 1482 (20.79%) | - |  | 822 (35.26%) | - |  | 660 (13.76%) | - |
| Adenocarcinoma | 4773 (66.97%) | - |  | 1304 (55.94%) | - |  | 3469 (72.33%) | - |
| Other | 872 (12.24%) | - |  | 205 (8.79%) | - |  | 667 (13.91%) | - |

**Supplementary Table 2.** Associations of height-associated variants and lung cancer risk in NJMU and FLCCA studies

| **NO.** | **CHR** | **SNP** | **Gene** | **Height association** ^b^ | | | **Minor/**  **Major** ^c^ | **MAF** ^c^ | **NJMU** ^d^ | |  | **FLCCA** ^e^ | | **Het *P*** ^f^ |
| --- | --- | --- | --- | --- | --- | --- | --- | --- | --- | --- | --- | --- | --- | --- |
|  |  |  |  | **E/O** ^a^ | **β** | **SE** |  |  | **OR(95%CI)** | ***P*** |  | **OR(95%CI)** | ***P*** |  |
| 1 | 1 | rs2284746 | *MFAP2* | C/G | 0.057 | 0.006 | G/C | 0.25 | 0.99(0.90,1.08) | 8.06E-01 |  | 0.94(0.88,1.00) | 5.39E-02 | 3.46E-01 |
| 2 | 1 | rs7513464 | *SPAG17* | C/G | 0.058 | 0.005 | C/G | 0.35 | 0.91(0.83,0.99) | 3.76E-02 |  | 1.06(0.99,1.13) | 7.63E-02 | 6.48E-03 |
| 3 | 1 | rs11205277 | *SV2A* | A/G | 0.044 | 0.006 | G/A | 0.29 | 1.05(0.96,1.15) | 2.54E-01 |  | 0.98(0.92,1.04) | 5.36E-01 | 1.98E-01 |
| 4 | 1 | rs1325598 | *PAPPA2* | A/G | 0.037 | 0.006 | A/G | 0.24 | 1.02(0.92,1.13) | 6.75E-01 |  | 0.99(0.92,1.06) | 7.10E-01 | 5.77E-01 |
| 5 | 1 | rs1926872 | *GLT25D2* | T/C | 0.046 | 0.005 | C/T | 0.45 | 1.04(0.95,1.13) | 3.85E-01 |  | 1.04(0.98,1.11) | 1.99E-01 | 9.52E-01 |
| 6 | 1 | rs4472734 | *PTPN14* | T/C | 0.028 | 0.005 | C/T | 0.49 | 1.00(0.92,1.09) | 9.72E-01 |  | 1.02(0.96,1.08) | 5.58E-01 | 7.08E-01 |
| 7 | 1 | rs1890995 | *TGFB2* | A/G | 0.039 | 0.005 | G/A | 0.30 | 0.96(0.87,1.06) | 4.19E-01 |  | 1.00(0.94,1.07) | 9.60E-01 | 4.93E-01 |
| 8 | 2 | rs4665736 | *RBJ* | T/C | 0.029 | 0.006 | C/T | 0.48 | 1.09(1.00,1.18) | 5.16E-02 |  | 0.93(0.88,0.99) | 2.89E-02 | 4.25E-03 |
| 9 | 2 | rs11694842 | *DNMT3A* | A/G | 0.053 | 0.008 | G/A | 0.09 | 1.05(0.90,1.23) | 5.24E-01 |  | 0.99(0.88,1.10) | 8.20E-01 | 5.15E-01 |
| 10 | 2 | rs3769528 | *LTBP1* | A/G | 0.060 | 0.006 | G/A | 0.16 | 1.12(1.01,1.25) | 3.70E-02 |  | 0.98(0.91,1.07) | 6.81E-01 | 5.49E-02 |
| 11 | 2 | rs3755206 | *CRIM1* | T/G | 0.046 | 0.007 | G/T | 0.18 | 0.95(0.85,1.05) | 3.04E-01 |  | 0.96(0.88,1.04) | 2.83E-01 | 8.57E-01 |
| 12 | 2 | rs3771381 | *ZNF638* | A/T | 0.041 | 0.005 | A/T | 0.46 | 0.92(0.85,1.01) | 6.71E-02 |  | 1.00(0.94,1.06) | 9.89E-01 | 1.35E-01 |
| 13 | 2 | rs867529 | *EIF2AK3* | C/G | 0.036 | 0.005 | C/G | 0.49 | 1.05(0.96,1.15) | 2.77E-01 |  | 1.03(0.97,1.10) | 2.76E-01 | 7.82E-01 |
| 14 | 2 | rs1351164 | *DIRC3* | T/C | 0.021 | 0.005 | T/C | 0.41 | 0.96(0.88,1.04) | 3.03E-01 |  | 1.01(0.95,1.08) | 6.91E-01 | 2.85E-01 |
| 15 | 2 | rs7588654 | *NHEJ1* | T/C | 0.047 | 0.005 | C/T | 0.48 | 0.96(0.88,1.04) | 3.11E-01 |  | 1.02(0.96,1.08) | 5.14E-01 | 2.29E-01 |
| 16 | 2 | rs6728302 | *DIS3L2* | A/G | 0.065 | 0.005 | G/A | 0.48 | 1.00(0.92,1.09) | 9.46E-01 |  | 0.98(0.92,1.04) | 5.34E-01 | 6.74E-01 |
| 17 | 3 | rs6772112 | *VGLL4* | T/C | 0.035 | 0.006 | C/T | 0.11 | 0.92(0.81,1.05) | 2.08E-01 |  | 1.07(0.98,1.18) | 1.40E-01 | 5.90E-02 |
| 18 | 3 | rs13072744 | *RYBP* | T/C | 0.034 | 0.006 | T/C | 0.48 | 1.01(0.93,1.10) | 8.75E-01 |  | 1.06(1.00,1.13) | 4.56E-02 | 2.93E-01 |
| 19 | 3 | rs7636293 | *C3orf47* | T/C | 0.035 | 0.005 | C/T | 0.34 | 1.01(0.92,1.10) | 8.39E-01 |  | 1.00(0.94,1.07) | 9.95E-01 | 8.72E-01 |
| 20 | 3 | rs6763931 | *ZBTB38* | A/G | 0.059 | 0.005 | A/G | 0.33 | 1.08(0.99,1.18) | 6.66E-02 |  | 0.99(0.93,1.05) | 7.44E-01 | 9.48E-02 |
| 21 | 3 | rs9818941 | *SHOX2* | A/G | 0.024 | 0.005 | A/G | 0.43 | 0.99(0.91,1.08) | 9.05E-01 |  | 0.96(0.91,1.02) | 2.16E-01 | 5.30E-01 |
| 22 | 3 | rs4243400 | *FNDC3B* | A/G | 0.045 | 0.005 | A/G | 0.45 | 1.06(0.97,1.15) | 1.81E-01 |  | 0.98(0.92,1.04) | 4.75E-01 | 1.33E-01 |
| 23 | 3 | rs572169 | *GHSR* | T/C | 0.025 | 0.005 | T/C | 0.42 | 1.06(0.98,1.15) | 1.61E-01 |  | 0.99(0.93,1.05) | 7.62E-01 | 1.91E-01 |
| 24 | 4 | rs13131350 | *LCORL* | A/G | 0.085 | 0.006 | G/A | 0.26 | 0.93(0.85,1.03) | 1.60E-01 |  | 0.97(0.90,1.04) | 4.12E-01 | 5.19E-01 |
| 25 | 4 | rs2227901 | *REST* | A/G | 0.029 | 0.005 | A/G | 0.37 | 0.95(0.87,1.03) | 2.12E-01 |  | 1.00(0.94,1.06) | 9.24E-01 | 3.39E-01 |
| 26 | 4 | rs16848425 | *ADAMTS3* | T/C | 0.039 | 0.006 | T/C | 0.26 | 1.02(0.92,1.13) | 7.69E-01 |  | 1.03(0.96,1.11) | 4.12E-01 | 8.09E-01 |
| 27 | 4 | rs2011962 | *RASGEF1B* | A/C | 0.047 | 0.006 | C/A | 0.17 | 0.95(0.85,1.06) | 3.81E-01 |  | 1.01(0.93,1.09) | 8.10E-01 | 3.92E-01 |
| 28 | 4 | rs2454206 | *TET2* | A/G | 0.032 | 0.006 | G/A | 0.19 | 0.97(0.87,1.07) | 5.15E-01 |  | 0.92(0.85,0.99) | 1.84E-02 | 4.06E-01 |
| 29 | 4 | rs6845999 | *HHIP* | T/C | 0.042 | 0.006 | T/C | 0.2 | 0.97(0.88,1.07) | 5.74E-01 |  | 0.97(0.90,1.04) | 3.91E-01 | 9.48E-01 |
| 30 | 5 | rs6180 | *GHR* | A/C | 0.022 | 0.005 | A/C | 0.43 | 1.10(1.01,1.20) | 2.33E-02 |  | 1.00(0.94,1.07) | 9.16E-01 | 7.60E-02 |
| 31 | 5 | rs7704138 | *SLC38A9* | T/C | 0.031 | 0.005 | T/C | 0.46 | 1.02(0.94,1.11) | 6.38E-01 |  | 0.98(0.92,1.04) | 5.38E-01 | 4.57E-01 |
| 32 | 5 | rs7708474 | *CEP120* | T/G | 0.028 | 0.005 | G/T | 0.36 | 0.95(0.87,1.03) | 2.13E-01 |  | 0.97(0.91,1.04) | 3.71E-01 | 6.34E-01 |
| 33 | 5 | rs4282339 | *SLIT3* | A/G | 0.031 | 0.006 | A/G | 0.19 | 1.01(0.90,1.13) | 8.71E-01 |  | 1.06(0.98,1.15) | 1.59E-01 | 4.92E-01 |
| 34 | 5 | rs12153391 | *CTB-78H18.1* | A/C | 0.022 | 0.005 | A/C | 0.43 | 0.93(0.85,1.01) | 8.34E-02 |  | 0.99(0.93,1.05) | 8.00E-01 | 2.11E-01 |
| 35 | 5 | rs889014 | *STC2-BOD1* | T/C | 0.039 | 0.007 | T/C | 0.13 | 1.02(0.89,1.16) | 7.67E-01 |  | 0.98(0.89,1.08) | 7.00E-01 | 6.41E-01 |
| 36 | 6 | rs3812163 | *BMP6* | A/T | 0.03 | 0.006 | T/A | 0.24 | 1.02(0.93,1.13) | 6.46E-01 |  | 1.00(0.93,1.08) | 9.47E-01 | 7.43E-01 |
| 37 | 6 | rs806794 | *HIST1H2BF* | A/G | 0.075 | 0.006 | A/G | 0.24 | 1.13(1.02,1.24) | 1.42E-02 |  | 0.96(0.89,1.03) | 2.36E-01 | 7.69E-03 |
| 38 | 6 | rs11970475 | *UBD* | A/G | 0.063 | 0.006 | A/G | 0.15 | 1.04(0.93,1.16) | 4.92E-01 |  | 0.96(0.87,1.06) | 4.53E-01 | 3.11E-01 |
| 39 | 6 | rs6918981 | *NUDT3* | A/G | 0.070 | 0.006 | G/A | 0.17 | 1.13(1.00,1.27) | 4.99E-02 |  | 1.09(1.00,1.18) | 5.42E-02 | 6.10E-01 |
| 40 | 6 | rs9472414 | *SUPT3H* | A/T | 0.024 | 0.005 | A/T | 0.26 | 0.98(0.90,1.08) | 7.25E-01 |  | 0.98(0.92,1.05) | 6.19E-01 | 9.91E-01 |
| 41 | 6 | rs7759938 | *LIN28B* | T/C | 0.028 | 0.005 | C/T | 0.31 | 0.97(0.89,1.07) | 5.50E-01 |  | 1.04(0.98,1.11) | 2.10E-01 | 2.23E-01 |
| 42 | 6 | rs1415701 | *L3MBTL3* | A/G | 0.053 | 0.005 | A/G | 0.38 | 0.95(0.87,1.03) | 2.01E-01 |  | 0.99(0.93,1.05) | 6.63E-01 | 4.45E-01 |
| 43 | 6 | rs6570507 | *GPR126* | A/G | 0.034 | 0.005 | A/G | 0.40 | 0.94(0.86,1.02) | 1.58E-01 |  | 0.98(0.92,1.04) | 4.89E-01 | 4.66E-01 |
| 44 | 7 | rs7777484 | *GNA12* | A/G | 0.058 | 0.006 | G/A | 0.23 | 1.02(0.92,1.13) | 7.68E-01 |  | 0.94(0.87,1.01) | 8.70E-02 | 2.15E-01 |
| 45 | 7 | rs4470914 | *TWISTNB* | T/C | 0.037 | 0.006 | T/C | 0.2 | 1.08(0.97,1.19) | 1.54E-01 |  | 0.99(0.92,1.06) | 7.14E-01 | 1.73E-01 |
| 46 | 7 | rs6959212 | *SFRP4* | T/C | 0.025 | 0.005 | T/C | 0.31 | 0.98(0.90,1.07) | 6.91E-01 |  | 0.99(0.93,1.06) | 7.99E-01 | 8.60E-01 |
| 47 | 8 | rs10448080 | *EXTL3* | T/C | 0.029 | 0.005 | C/T | 0.35 | 0.98(0.90,1.07) | 6.91E-01 |  | 1.01(0.95,1.08) | 6.82E-01 | 5.73E-01 |
| 48 | 8 | rs13273123 | *PLAG1* | A/G | 0.103 | 0.009 | G/A | 0.09 | 0.92(0.77,1.10) | 3.65E-01 |  | 0.87(0.77,0.98) | 1.77E-02 | 5.83E-01 |
| 49 | 8 | rs4733789 | *MYC* | T/C | 0.030 | 0.005 | T/C | 0.39 | 1.04(0.95,1.13) | 4.07E-01 |  | 0.94(0.89,1.00) | 6.44E-02 | 8.00E-02 |
| 50 | 8 | rs2062078 | *GSDMC* | T/G | 0.035 | 0.005 | G/T | 0.28 | 1.05(0.96,1.16) | 2.58E-01 |  | 1.00(0.94,1.07) | 9.57E-01 | 3.72E-01 |
| 51 | 8 | rs733254 | *ZFAT* | C/A | 0.023 | 0.005 | C/A | 0.45 | 0.99(0.91,1.08) | 8.77E-01 |  | 1.00(0.94,1.06) | 9.93E-01 | 9.04E-01 |
| 52 | 9 | rs10512248 | *PTCH1* | T/G | 0.027 | 0.005 | G/T | 0.32 | 1.01(0.93,1.11) | 7.45E-01 |  | 1.01(0.95,1.08) | 6.65E-01 | 9.95E-01 |
| 53 | 9 | rs10858250 | *QSOX2* | A/G | 0.052 | 0.005 | G/A | 0.25 | 1.01(0.91,1.11) | 8.97E-01 |  | 0.98(0.91,1.05) | 5.79E-01 | 6.66E-01 |
| 54 | 10 | rs779933 | *ZMIZ1* | A/G | 0.026 | 0.005 | A/G | 0.26 | 0.95(0.86,1.03) | 2.22E-01 |  | 1.01(0.94,1.08) | 7.69E-01 | 2.47E-01 |
| 55 | 11 | rs606452 | *SERPINH1* | A/C | 0.039 | 0.005 | A/C | 0.48 | 0.97(0.89,1.05) | 4.45E-01 |  | 1.03(0.97,1.10) | 3.23E-01 | 2.29E-01 |
| 56 | 11 | rs11021504 | *MAML2* | A/T | 0.083 | 0.015 | T/A | 0.47 | 0.99(0.91,1.07) | 8.05E-01 |  | 0.94(0.89,1.00) | 4.63E-02 | 3.28E-01 |
| 57 | 11 | rs2510897 | *DDX6* | A/C | 0.029 | 0.005 | C/A | 0.37 | 1.10(1.02,1.20) | 2.00E-02 |  | 1.05(0.99,1.12) | 1.05E-01 | 3.64E-01 |
| 58 | 12 | rs1971762 | *ATP5G2* | T/C | 0.041 | 0.005 | T/C | 0.37 | 1.08(1.00,1.18) | 6.14E-02 |  | 1.08(1.01,1.15) | 1.59E-02 | 9.32E-01 |
| 59 | 12 | rs3809128 | *PAN2* | T/C | 0.089 | 0.007 | T/C | 0.16 | 1.01(0.91,1.11) | 9.19E-01 |  | 0.99(0.91,1.07) | 7.55E-01 | 7.85E-01 |
| 60 | 12 | rs2066808 | *STAT2* | A/G | 0.081 | 0.012 | G/A | 0.04 | 1.01(0.77,1.31) | 9.54E-01 |  | 1.11(0.90,1.36) | 3.23E-01 | 5.75E-01 |
| 61 | 12 | rs1042725 | *HMGA2* | T/C | 0.027 | 0.006 | C/T | 0.18 | 1.05(0.95,1.16) | 3.52E-01 |  | 1.00(0.93,1.08) | 9.13E-01 | 4.96E-01 |
| 62 | 12 | rs10748128 | *RP11-956E11.1* | T/G | 0.027 | 0.006 | G/T | 0.36 | 1.09(0.99,1.19) | 7.00E-02 |  | 0.94(0.88,1.00) | 4.66E-02 | 8.49E-03 |
| 63 | 12 | rs11107116 | *SOCS2* | T/G | 0.035 | 0.005 | T/G | 0.28 | 1.05(0.96,1.15) | 3.03E-01 |  | 1.01(0.95,1.08) | 7.70E-01 | 5.06E-01 |
| 64 | 12 | rs7313075 | *IGF1* | A/C | 0.073 | 0.005 | A/C | 0.30 | 0.98(0.89,1.08) | 7.19E-01 |  | 1.01(0.95,1.08) | 6.80E-01 | 5.94E-01 |
| 65 | 13 | rs3118905 | *DLEU1* | A/G | 0.083 | 0.018 | A/G | 0.02 | 1.13(0.84,1.54) | 4.23E-01 |  | 1.13(0.91,1.41) | 2.60E-01 | 9.93E-01 |
| 66 | 13 | rs7319045 | *GPC5* | A/G | 0.030 | 0.005 | G/A | 0.46 | 1.07(0.98,1.16) | 1.36E-01 |  | 0.99(0.93,1.05) | 7.35E-01 | 1.58E-01 |
| 67 | 14 | rs2093210 | *SIX6* | T/C | 0.028 | 0.006 | T/C | 0.21 | 1.08(0.98,1.19) | 1.26E-01 |  | 1.02(0.95,1.10) | 5.51E-01 | 3.80E-01 |
| 68 | 14 | rs699371 | *LTBP2* | T/C | 0.050 | 0.007 | T/C | 0.22 | 0.97(0.87,1.07) | 5.30E-01 |  | 1.02(0.95,1.10) | 6.13E-01 | 4.20E-01 |
| 69 | 14 | rs7158300 | *TRIP11* | T/C | 0.054 | 0.005 | T/C | 0.31 | 0.98(0.90,1.07) | 6.85E-01 |  | 1.03(0.97,1.10) | 3.75E-01 | 3.96E-01 |
| 70 | 15 | rs10519302 | *CYP19A1* | A/G | 0.057 | 0.005 | G/A | 0.32 | 1.00(0.92,1.10) | 9.37E-01 |  | 1.02(0.96,1.09) | 4.69E-01 | 7.13E-01 |
| 71 | 15 | rs7184046 | *PTPN9* | C/G | 0.032 | 0.005 | C/G | 0.34 | 1.04(0.96,1.14) | 3.46E-01 |  | 1.02(0.95,1.08) | 5.97E-01 | 6.55E-01 |
| 72 | 15 | rs2401171 | *ADAMTSL3* | T/G | 0.049 | 0.005 | T/G | 0.28 | 0.98(0.89,1.08) | 7.15E-01 |  | 1.01(0.94,1.09) | 7.26E-01 | 6.16E-01 |
| 73 | 15 | rs3817428 | *ACAN* | C/G | 0.052 | 0.008 | G/C | 0.12 | 0.99(0.87,1.13) | 9.22E-01 |  | 1.12(1.02,1.24) | 2.34E-02 | 1.50E-01 |
| 74 | 15 | rs2871865 | *IGF1R* | C/G | 0.094 | 0.009 | G/C | 0.11 | 0.96(0.82,1.12) | 5.73E-01 |  | 0.94(0.84,1.05) | 2.51E-01 | 8.42E-01 |
| 75 | 15 | rs2573652 | *ADAMTS17* | T/C | 0.035 | 0.005 | T/C | 0.44 | 1.02(0.94,1.11) | 6.52E-01 |  | 0.97(0.91,1.03) | 2.84E-01 | 3.19E-01 |
| 76 | 16 | rs11648796 | *NARFL* | A/G | 0.045 | 0.008 | A/G | 0.23 | 0.90(0.81,1.00) | 4.28E-02 |  | 0.96(0.88,1.04) | 3.12E-01 | 3.42E-01 |
| 77 | 16 | rs1659127 | *MKL2-PARN* | A/G | 0.032 | 0.006 | G/A | 0.44 | 1.05(0.97,1.14) | 2.53E-01 |  | 1.01(0.95,1.07) | 7.24E-01 | 4.72E-01 |
| 78 | 16 | rs258324 | *CDK10* | T/G | 0.056 | 0.006 | T/G | 0.30 | 0.99(0.90,1.08) | 7.54E-01 |  | 0.95(0.89,1.02) | 1.66E-01 | 5.80E-01 |
| 79 | 17 | rs2270518 | *KDM6B* | T/C | 0.039 | 0.006 | T/C | 0.17 | 1.00(0.91,1.11) | 9.49E-01 |  | 0.96(0.89,1.03) | 2.91E-01 | 4.91E-01 |
| 80 | 17 | rs4986172 | *ACBD4* | T/C | 0.024 | 0.005 | T/C | 0.47 | 0.99(0.91,1.08) | 8.98E-01 |  | 1.01(0.95,1.08) | 6.89E-01 | 7.33E-01 |
| 81 | 17 | rs4794665 | *C17orf67* | A/G | 0.025 | 0.006 | A/G | 0.20 | 1.08(0.96,1.21) | 2.07E-01 |  | 1.02(0.94,1.11) | 6.17E-01 | 4.59E-01 |
| 82 | 17 | rs757608 | *TBX4* | A/G | 0.044 | 0.005 | A/G | 0.30 | 0.96(0.88,1.05) | 3.56E-01 |  | 0.96(0.89,1.02) | 1.81E-01 | 9.56E-01 |
| 83 | 17 | rs3785574 | *MAP3K3* | T/C | 0.036 | 0.005 | C/T | 0.49 | 0.97(0.89,1.05) | 4.43E-01 |  | 1.01(0.95,1.07) | 7.99E-01 | 4.41E-01 |
| 84 | 18 | rs4369779 | *CABLES1* | T/C | 0.061 | 0.006 | T/C | 0.14 | 1.11(0.99,1.23) | 6.75E-02 |  | 1.05(0.97,1.13) | 2.23E-01 | 4.41E-01 |
| 85 | 18 | rs11082671 | *U7* | G/A | 0.107 | 0.019 | A/G | 0.23 | 1.11(1.00,1.22) | 4.88E-02 |  | 1.00(0.92,1.07) | 9.11E-01 | 9.92E-02 |
| 86 | 18 | rs16950303 | *DYM* | A/G | 0.041 | 0.005 | G/A | 0.27 | 1.01(0.92,1.11) | 7.56E-01 |  | 0.95(0.89,1.02) | 1.65E-01 | 2.85E-01 |
| 87 | 18 | rs17782313 | *MC4R* | T/C | 0.031 | 0.006 | C/T | 0.19 | 1.13(1.02,1.25) | 2.06E-02 |  | 0.98(0.91,1.05) | 5.25E-01 | 2.50E-02 |
| 88 | 19 | rs7250071 | *ILF3* | T/C | 0.040 | 0.005 | C/T | 0.36 | 0.97(0.89,1.05) | 4.28E-01 |  | 1.06(0.99,1.13) | 7.65E-02 | 9.10E-02 |
| 89 | 20 | rs2145272 | *BMP2* | A/G | 0.062 | 0.008 | G/A | 0.11 | 1.03(0.91,1.17) | 6.14E-01 |  | 0.98(0.89,1.07) | 6.06E-01 | 4.75E-01 |
| 90 | 20 | rs6060369 | *UQCC* | T/C | 0.060 | 0.005 | C/T | 0.28 | 0.93(0.85,1.02) | 1.25E-01 |  | 0.98(0.91,1.05) | 5.27E-01 | 3.86E-01 |
| 91 | 20 | rs6030712 | *RBL1* | A/G | 0.032 | 0.005 | A/G | 0.50 | 0.94(0.87,1.02) | 1.62E-01 |  | 1.03(0.97,1.09) | 4.13E-01 | 5.13E-01 |
| 92 | 22 | rs115295339 | *SYN3* | T/C | 0.028 | 0.006 | T/C | 0.28 | 1.05(0.95,1.15) | 3.35E-01 |  | 0.95(0.88,1.01) | 1.08E-01 | 8.52E-02 |
| SNPs failed in pleiotropic effect test in NJMU study | | | | | | | | | | | | | | |
| 93 | 1 | rs12410416g | *DNM3* | T/C | 0.031 | 0.005 | C/T | 0.27 | 1.14(1.04,1.24) | 5.06E-03 |  | 1.07(1.01,1.15) | 3.06E-02 | 3.28E-01 |
| 94 | 2 | rs3791675g | *EFEMP1* | T/C | 0.070 | 0.006 | C/T | 0.24 | 1.19(1.07,1.32) | 8.72E-04 |  | 1.05(0.97,1.13) | 2.13E-01 | 4.67E-02 |
| 95 | 2 | rs611203g | *PLCD4* | A/G | 0.035 | 0.006 | G/A | 0.22 | 1.13(1.02,1.24) | 1.46E-02 |  | 1.06(0.99,1.14) | 1.09E-01 | 3.05E-01 |
| 96 | 6 | rs1865760g | *SLC17A2* | T/C | 0.037 | 0.005 | C/T | 0.30 | 1.14(1.04,1.24) | 5.32E-03 |  | 0.98(0.91,1.05) | 4.98E-01 | 8.52E-03 |
| 97 | 11 | rs174547g | *FADS1* | T/C | 0.042 | 0.005 | T/C | 0.43 | 1.20(1.10,1.30) | 4.27E-05 |  | 1.08(1.02,1.16) | 1.39E-02 | 7.27E-02 |
| 98 | 11 | rs1938679g | *CCND1* | T/C | 0.033 | 0.005 | T/C | 0.38 | 0.89(0.82,0.97) | 7.26E-03 |  | 1.03(0.97,1.10) | 3.21E-01 | 5.93E-03 |
| SNPs failed in pleiotropic effect test in FLCCA study | | | | | | | | | | | | | | |
| 99 | 5 | rs10037512h | *MEF2C* | T/C | 0.031 | 0.005 | T/C | 0.40 | 1.07(0.99,1.17) | 9.29E-02 |  | 1.09(1.02,1.16) | 7.56E-03 | 8.15E-01 |
| 100 | 5 | rs537930h | *CATSPER3* | T/G | 0.034 | 0.006 | T/G | 0.31 | 0.99(0.91,1.09) | 9.17E-01 |  | 1.10(1.03,1.18) | 5.00E-03 | 8.40E-02 |
| 101 | 11 | rs2237886h | *KCNQ1* | T/C | 0.037 | 0.007 | T/C | 0.19 | 0.98(0.88,1.10) | 7.49E-01 |  | 1.10(1.02,1.19) | 1.31E-02 | 9.23E-02 |

^a^ The effect allele associated with increased height from the published studies, E for effect allele, O for other allele;

^b^ The β estimates and standard errors (SE) of height-associated SNPs from the published studies;

^c^ Minor allele frequent of height-associated SNPs in 1000 Genomes Project Phase 3 East Asian population;

^d^ Results (OR, 95%CI and *P*) were derived from NJMU study and were adjusted for age, sex, pack-years, and first principal component;

^e^ Results (OR, 95%CI and *P*) were derived from FLCCA study and were adjusted for age and three principal components;

^f^ Heterogeneity test was conducted by meta-analysis of two studies.

**Supplementary Table 3.** Heterogeneity test showing pleiotropic effect of height-associated SNPs in two studies

|  | NJMU | |  | FLCCA | |
| --- | --- | --- | --- | --- | --- |
|  | SNPs | Pleiotropy *P* ^a^ |  | SNPs | Pleiotropy *P* ^a^ |
| All lung cancer | 101 | 6.15×10^-5^ |  | 101 | 4.25×10^-3^ |
| Lung adenocarcinoma | 101 | 8.57×10^-5^ |  | 101 | 0.011 |
| Lung squamous cell carcinoma | 101 | 0.019 |  | 101 | 0.110 |
| After filter test |  |  |  |  |  |
| All lung cancer | 95 | 0.068 |  | 98 | 0.061 |
| Lung adenocarcinoma | 95 | 0.024 |  | 98 | 0.099 |
| Lung squamous cell carcinoma | 95 | 0.246 |  | 98 | 0.179 |

^a^ Pleiotropic *P* value was tested by pleiotropy test in ‘gtx’ package in R.

**Supplementary Table 4.** Associations of genetic determined height and lung cancer risk in two studies

|  | NJMU ^a^ | |  | FLCCA ^b^ | | Het *P* ^c^ |
| --- | --- | --- | --- | --- | --- | --- |
|  | OR(95%CI) | *P* |  | OR(95%CI) | *P* |  |
| Lung adenocarcinoma |  |  |  |  |  |  |
| wGRS | 1.33 (1.03,1.71) | 0.026 |  | 1.12 (0.95,1.32) | 0.183 | 0.266 |
| IVW | 1.33 (1.04,1.72) | 0.025 |  | 1.12 (0.95,1.33) | 0.180 |  |
| Lung squamous cell carcinoma |  |  |  |  |  |  |
| wGRS | 1.26 (0.92,1.73) | 0.153 |  | 1.33 (0.98,1.80) | 0.070 | 0.821 |
| IVW | 1.26 (0.92,1.74) | 0.153 |  | 1.33 (0.98,1.81) | 0.070 |  |
| All lung cancer |  |  |  |  |  |  |
| wGRS | 1.31 (1.06,1.62) | 0.013 |  | 1.13 (0.97,1.32) | 0.114 | 0.276 |
| IVW | 1.32 (1.06,1.63) | 0.012 |  | 1.13 (0.97,1.32) | 0.110 |  |

^a^ Results (OR, 95%CI and *P*) were derived from NJMU study and were adjusted for age, sex, pack-years, and first principal component;

^b^ Results (OR, 95%CI and *P*) were derived from FLCCA study and were adjusted for age and three principal components;

^c^ Heterogeneity test was conducted by meta-analysis of two studies.

**Supplementary Figure**


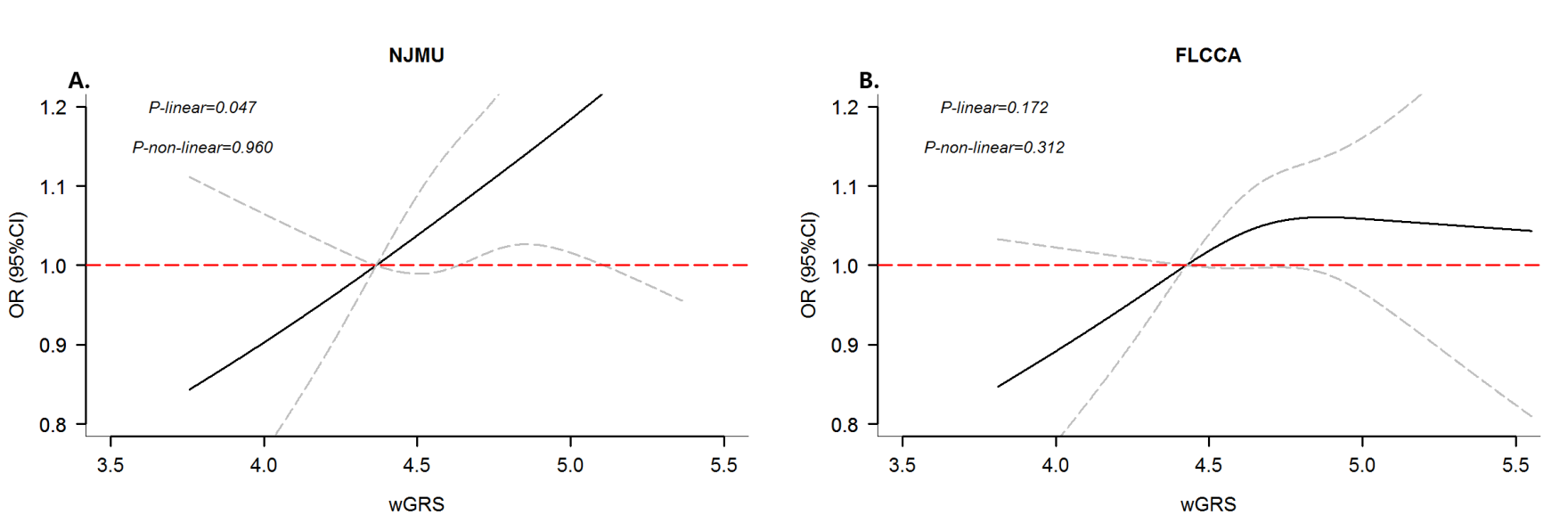


**Supplementary Figure 1.** Non-linear test between genetically increased height and lung cancer risk in NJMU (A) and FLCCA (B), based on restricted cubic spline function in the logistic regression model.
